# Investigating the effect of obsessive‐compulsive disorder on clinical symptoms of temporomandibular joint disorders

**DOI:** 10.1002/cre2.798

**Published:** 2023-10-15

**Authors:** Maryam‐Sadat Sadrzadeh‐Afshar, Behzad Salari, Ali shobeiri, Kimia HafeziMotlagh

**Affiliations:** ^1^ Department of Oral and Maxillofacial Medicine, Faculty of Dentistry AJA University of Medical Sciences Tehran Iran; ^2^ Orthodontics Department, Faculty of Dentistry, Tehran Medical Sciences Islamic Azad University Tehran Iran; ^3^ Department of Oral Medicine, School of Dentistry Tehran University of Medical Sciences Tehran Iran

**Keywords:** obsessive‐compulsive disorder, temporomandibular joint disorders, TMJ disease

## Abstract

**Objectives:**

Temporomandibular disorders (TMD) are a group of clinical conditions involving muscles of mastication, temporomandibular joint (TMJ), and related structures or both. TMD is characterized by facial pain in TMJ and muscles of mastication, limitation or deviation of jaw movement, and TMJ sounds during jaw movement and function. The highest risk of TMD prevalence is between 18 and 24 years, and a relationship is between chronic TMD and psychological disorders such as stress and depression.

The knowledge of the function of this joint and those with TMD symptoms when visiting the dentist will help to provide an ideal treatment plan for the patient. Therefore, if the therapist is familiar with the various etiological factors of this disorder, he will provide better treatment, especially if the simultaneous effect of psychological factors such as stress and *obsessive‐compulsive* disorder (OCD) along with occlusal factors such as posterior cross‐bite, overjet, and overbite is measured, it can be a valuable guide for clinicians.

**Methods and Materials:**

In this study, 385 patients were examined by DASS42 and *Maudsley's* test and classified into normal, with stress, and stress plus OCD groups. TMJ was examined for each of them by the TMD‐RDC test. The presence or absence of TMD was noted in their file.

**Results:**

The prevalence of TMD was 20.7% in the normal group, 30.70% in the stress group, and 44.68% in the stress and OCD group. After analyzing the data by SPSS 24 and performing analysis of variance and Duncan tests, no significant difference was found between the probability of TMD in normal and stressed groups, but the stress and OCD group has a higher chance of TMD.

**Conclusion:**

Although the co‐occurrence of stress and OCD is associated with the prevalence of TMD, it cannot be considered a cause of TMD.

## 1 INTRODUCTION

The temporomandibular joint (TMJ), including a complex set of the mandibular condyle and glenoid fossa of the temporal bone and its related structures, plays a crucial role in guiding mandibular movements and distributing stresses produced by daily activities such as chewing, swallowing, and speaking (Murphy et al., 2013). Temporomandibular disorders (TMD) are a group of musculoskeletal degenerative conditions associated with morphological and functional deformities. Among the common symptoms of TMD, we can mention pain, joint sounds, and limitation of range of motion (Tanaka et al., 2008). Temporomandibular pain and functional limitations are usually associated with many morbidities, including pain in other body muscles, including the neck and back. In developed countries, TMD is one of the most common chronic orofacial pain conditions, associated with negative impacts on individuals' quality of life (Montero et al., 2018). In addition to pain often aggravated by chewing or other functions, patients with TMD usually have irregular and limited jaw movements and report joint sounds (Tanaka et al., 2008).

TMD is a relatively common painful condition affecting up to 25% of the population, and its incidence peak is between the ages of 20 and 40 years. It is 1.5–2 times more common in women than men, and this difference is attributed to behavioral, hormonal, anatomic, and psychological factors (Lee et al., 2022). A recent systematic review and meta‐analysis reported that the prevalence of TMD is 31% in adults and 11% in children and adolescents (Valesan et al., 2021).

Some symptoms of TMD are clearly related to the joint, among which we can mention pain or sensitivity in TMJ and around the ear, limited mouth opening, and joint sounds during joint movement. In this case, the patient feels pain in front of the ear and recurring pain in the area of the head, neck, or shoulders (Li & Leung, 2021). The muscles' sensitivity of mastication may be caused by touching the muscle or while chewing, yawning, or pressing the teeth. Joint sounds, including clicking or joint crepitus, may be diagnosed during the joint examination. Also, problems related to joint function can be limited jaw opening or crooked jaw to one side when opening the mouth (List & Jensen, 2017). In some other cases, there may be a variety of problems that are indirectly related to the joint, such as depression, ear problems, swallowing disorders, headache, dizziness, and blurred vision associated with TMD (Wieckiewicz et al., 2015).

The etiology of TMD is multifactorial and is attributed to both physical and psychological factors. Physical factors are generally divided into two groups: arthrogenous, including joint internal disorders, and myogenous, referring to masticatory muscle disorders (Toh et al., 2021). What complicates the diagnosis and classification of TMD is that many patients refer with multiple diagnoses of TMD simultaneously. This disease cannot be attributed to a specific cause. Apart from physical factors, the relationship between psychosocial factors and TMD has also been described by researchers (Bitiniene et al., 2018). In TMD, as with other conditions that cause chronic pain, such as back pain and headache, it seems that those at risk of developing symptomatic TMD have similar psychological profiles and functional impairment (Ismail et al., 2016). High levels of depression and somatization have been associated with the incidence of TMD, and in addition, in those with TMD, symptoms may be aggravated during stressful events (Yap et al., 2002).

Obsessive‐compulsive disorder (OCD) is a type of mental disorder in which an individual needs to do daily routines many times, which is called compulsion, or has certain thoughts that are repeated (Goodman et al., 2021). OCD is considered the fourth most common psychiatric disorder and affects 2%–3% of the general population during their lifetime. Regarding the etiology of OCD, various things have been mentioned, such as physical and bodily changes, environment and behavioral habits, serotonin deficiency, family history, and stressful lifestyle (Adam et al., 2012). In OCD, anxiety‐provoking thoughts and fear are along with practical obsession. Such obsession is in such a way that an individual does to try to reduce his mental obsessions. These actions are repetitive, stereotyped, and even involuntary (Heyman, 2006). OCD has several subgroups, including obsession with doubt, obsession with revision, obsession with contamination, and obsession with repetition (Rapinesi et al., 2019).

Several studies have been conducted on the prevalence of OCD types. For example, Mohammadi et al. (2004) investigated the prevalence of OCD in Iran and reported that the prevalence of OCD was 1.8% (0.7% for men and 2.8% for women) (Mohammadi et al., 2004). Another article that measured the prevalence of OCD in one of Turkey's universities in 2009 reported the prevalence of OCD to be 2.4% and found a strong relationship between males living in government dormitories and living in student houses and verbal abuse in the family and OCD (Yoldascan et al., 2009).

According to the study results, almost one‐half to three‐quarters of those with OCD have at least one other disorder. Recent studies on comorbidity have concluded that 75% of adults with OCD and 25% of children with OCD experience major depressive disorder. The high rate of comorbidity has led some to conclude that obsession is another form of emotional disorder (Ricciardi & McNally, 1995). The highest rate of comorbidity of obsessive symptoms with depression and anxiety disorders is observed. The comorbidity of anxiety disorder is common before and after an individual is diagnosed with OCD, and the risk of developing depression, anxiety, eating disorders, and nervous tics increases during an individual's OCD diagnosis (Yaryura‐Tobias et al., 2000). In fact, it may be possible to conclude that OCD is a disorder that includes a wide spectrum of psychiatric symptoms.

Since, according to the literature review, some of the factors affecting TMD are in the field of mental diseases, and on the other hand, the effect of OCD on TMD has not been investigated, this study was conducted to investigate the effect of OCD on TMD.

## 2 MATERIALS AND METHODS

This project has been approved by the Ethics Committee of the Army University of Medical Sciences University No. IR.AJAUMS.REC.1401.041.

To determine the sample size in this study using the Cochran formula, the sample size is *n* = 385 with an error rate of 5% and 95% confidence interval. The subjects were selected among those referred to medical centers in Tehran and Mashhad. For this purpose, questionnaires were given to the patients randomly, and then a physician examined them in terms of TMJ. To increase the accuracy of measurements and examinations, all the stages have been performed twice to achieve maximum accuracy. The examiner should have enough experience, skills, and knowledge of the study process and the method of performing the tests and the examinations accurately in the presence of skilled professors. The process of the questionnaires and the reason for conducting this study were fully explained to the patients, and participation in this study was completely voluntary.

Due to the high prevalence of OCD along with stress and the inability to find a pure OCD group, we examine OCD and stress together in one group. Also, to compare different groups, based on the initial sample of *n* = 130, the number of people in the healthy (normal) groups, those with only stress, and those with stress and OCD were 67, 47, and 16 people, respectively. Based on this initial grouping, the ratio of each of the above groups in the population was estimated, and the sample size of each group was calculated (Table 1).

**Table 1:** Allocation of the sample size between the study groups.

Number	Ratio	Group
198	0.515	Normal
140	0.364	Stress only
47	0.121	Stress and OCD
385	Total	

Abbreviation: OCD, obsessive‐compulsive disorder.

The study inclusion criteria include having class (I), and normal overjet and overbite, and the study exclusion criteria include a history of trauma to TMJ, craniofacial syndromes, cross‐bite, and clear occlusal interference.

First, the questionnaires were given to the subjects, and after the completion, the TMJ of each individual was examined. Before the examination, each patient was examined for the study inclusion criteria. In the case of the study exclusion criteria, while evaluating the patient's cooperation in completing the questionnaires by explaining the reason for withdrawal from the study, the individual was left out of the study. The individual who had the study inclusion criteria was examined.

In this study, for the presence or absence of clinical symptoms of TMD, a detailed examination of TMJ was based on the RCD‐TMD questionnaire. If the presence of TMD was confirmed, its symptoms were recorded in a special form for each patient. A dental caliper was used for the accurate numerical values in the examinations, including the maximum overjet.

The *Maudsley* Obsessive Compulsive Inventory (MOCI) is designed with 30 yes and no questions. The DASS‐42 stress questionnaire is designed in such a way that the questions have 4 options and are completed as self‐assessments. The range of answers varies from “never” to “always,” so that people can give their answers as one of the options in front of the corresponding question as “never,” “a little,” “sometimes,” and “always” with a cross mark. Scoring is from “zero” to “three,” and a score of zero is considered for the “never” option, a score of one for the “a little” option, a score of two for the “sometimes” option, and a score of three for the “always” option.

MOCI with 30 questions measures four components: checking, cleaning, repetition, and doubt. Depression Anxiety and Stress Scale (DASS42) measures an individual's stress with 42 questions, including 14 questions about individuals' stress, 14 questions about anxiety, and 14 questions about depression.

## 3 STATISTICAL ANALYSIS

This study used descriptive and inferential statistics to analyze data obtained from the sample. First, each variable was described in the form of tables and statistical indicators, and then SPSS was used to test the hypotheses. In inferential statistics, correlation analysis, logistic regression model, and independent two‐sample *t* test were used.

## 4 RESULTS

At the beginning of the research questionnaire, questions were collected to obtain the demographic information, including the general information of the participants in this study, the results of which are given below. Table 2 shows a summary of the frequency of each of the studied variables in the studied groups. To investigate the interaction between the variables, based on the measurement scale of the variables, one of the bi‐serial correlation coefficients, Spearman's correlation coefficient and/or Pearson's correlation coefficient, was used.

**Table 2:** Frequency percentage of each variable in the studied groups.

Percentage	Frequency	Variable	
55.8	215	Male	Sex
44.2	170	Female	
2.9	11	Elementary	Education
4.9	19	High school	
29.6	114	Diploma	
62.6	241	University	
63.6	245	Normal	Depression level
13.5	52	Mild	
17.9	69	Moderate	
4.9	19	Severe	
61.3	236	Normal	Anxiety level
22.3	86	Mild	
10.9	42	Moderate	
4.7	18	Severe	
0.8	3	Very severe	
51.4	198	Normal	Stress level
21.8	84	Mild	
14.8	57	Moderate	
11.4	44	Severe	
0.5	2	Very severe	
90.0	350	No pain	Myofascial pain
7	27	With pain	
2.1	8	Myofascial pain with opening limitation	
73.8	284	Without displacement	Disc displacement
23.9	92	Displacement with reduction	
1.8	7	Displacement without reduction with opening limitation	
0.5	2	Displacement without reduction and without opening limitation	
90.4	384	No degenerative disease	Degenerative joint disorders
3.4	13	Arthralgia	
5.5	21	Osteoarthritis	
0.8	3	Osteoarthrosis	
92.7	357	No pain	Degree of chronic pain
5.5	21	Grade l	
1.8	7	Grade ll	
72.7	280	Yes	TMD
27.3	105	No	
17		Min	Age
62		Max	
34.17		Mean	
10.867		Standard deviation	
0.07		Min	OCD
0.83		Max	
0.3558		Mean	
0.14702		Standard deviation	

Abbreviation: OCD, obsessive‐compulsive disorder.

### 4.1 Education level

The correlation coefficient result between the level of education and other research variables in Table 3 shows a significant relationship between the level of education and age only (*p* < .05).

**Table 3:** Statistical relationship of each of the studied variables with others.

TMD	Chronic pain	Degenerative joint disorders	Disc displacement	Myofascial pain	OCD	Stress level	Anxiety level	Depression level	Education	Age	Sex		
−.021	−.044	−.088	−.070	−.080	.039	−.042	.006	−.036		.147	−.089	Correlation coefficients	Education
.687	.393	.085	.170	.117	.449	.408	.914	.485		.004	.080	*p* Value	
.302	.261	.213	.339	.320	.523	.634	.593		−.036	.175	.194	Correlation coefficients	Depression level
.000	.000	.000	.000	.000	.000	.000	.000		.485	.001	.000	*p* Value	
.145	.195	0.099	160	165	430	.613		.593	.006	.030	.148	Correlation coefficients	Anxiety level
.004	.000	.053	.002	.001	.000	.000		.000	.914	.555	.004	*p* Value	
.168	.212	.146	.207	.202	.687		.613	.634	.042	.134	.116	Correlation coefficients	Stress level
.001	.000	.004	.000	.000	.000		.000	.000	.408	.009	.023	*p* Value	
.147	.245	.220	.153	.250		.687	.430	.523	.039	.044	.092	Correlation coefficients	OCD
.004	.000	.000	.003	.000		.000	.000	.000	.449	.388	.072	*p* Value	
486	784	738	.615		.250	.202	.165	.320	−.080	.103	.070	Correlation coefficients	Myofascial pain
.000	.000	.000	.000		.000	.000	.001	.000	.117	.043	.171	*p* Value	
.908	.544	.479		.615	153	.207	.160	.339	−.070	.206	.115	Correlation coefficients	Disc displacement
.000	.000	.000		.000	.003	.000	.002	.000	.170	.000	.024	*p* Value	
.500	.682		.479	.738	.220	.146	.099	.213	−.088	.097	.075	Correlation coefficients	Degenerative joint disorders
.000	.000		.000	.000	.000	.004	.053	.000	.085	.058	.144	*p* Value	
.430		.682	.544	.784	.245	.212	.195	.261	−.044	110	.084	Correlation coefficients	Chronic pain
.000		.000	.000	.000	.000	.000	.000	.000	.393	.030	.099	*p* Value	
	.430	.500	.908	.486	.147	.168	.145	.302	−.021	191	.137	Correlation coefficients	TMD
	.000	.000	.000	.000	.004	.001	.004	.000	.687	.000	.007	*p* Value	

Abbreviation: OCD, obsessive‐compulsive disorder.

### 4.2 Depression level

The result of the correlation coefficient of the variable of depression level and other research variables in Table 3 shows a significant difference between the depression level and the variables of gender, age, anxiety level, stress level, practical obsession, muscle pain, disc displacement, degenerative joint diseases, classified chronic pain, and TMD (*p* < .05).

### 4.3 Anxiety level

The result of the correlation coefficient of the variable of anxiety level and other research variables in Table 3 shows a significant relationship between the anxiety level and the variables of gender, depression level, stress level, OCD, muscle pain, disc displacement, classified chronic pain, and TMD (*p* < .05).

### 4.4 Stress level

The result of the correlation coefficient of the variable of stress level and other research variables in Table 3 shows a significant difference between the stress level and the variables of gender, age, depression level, anxiety level, OCD, facial muscle pain, disc displacement, degenerative joint diseases, classified chronic pain, and TMD (*p* < .05).

### 4.5 OCD

The result of the correlation coefficient of the variable of OCD and other research variables in Table 3 shows a significant relationship between OCD and the variables of depression level, anxiety level, stress level, facial muscle pain, disc displacement, degenerative joint diseases, classified chronic pain, and TMD (*p* < .05).

### 4.6 Facial muscle pain

The result of the correlation coefficient of the facial muscle pain variable and other research variables in Table 3 shows a significant relationship between facial muscle pain and variables of age, depression level, anxiety level, stress level, OCD, disc displacement, degenerative joint diseases, classified chronic pain, and TMD (*p* < .05).

### 4.7 Disc displacement

The result of the correlation coefficient of the variable of disc displacement and other research variables in Table 3 shows a significant relationship between the disc displacement and the variables of gender, age, depression level, anxiety level, stress level, OCD, facial muscle pain, disc displacement, degenerative joint diseases, classified chronic pain, and TMD (*p* < .05).

### 4.8 Degenerative joint disorders

The result of the correlation coefficient of the variable of degenerative joint diseases and other research variables in Table 3 shows a significant relationship between degenerative joint diseases and the variables of depression level, stress level, OCD, facial muscle pain, disc displacement, classified chronic pain, and TMD (*p* < .05).

### 4.9 Classified chronic pain

The result of the correlation coefficient of the variable of classified chronic pain and other research variables in Table 3 shows a significant relationship between classified chronic pain and variables of age, depression level, anxiety level, stress level, OCD, facial muscle pain, disc displacement, degenerative joint diseases, and TMD (*p* < .05).

### 4.10 TMD

The result of the correlation coefficient of the variable of TMD and other research variables in Table 3 shows a significant relationship between TMD and the variables of gender, age, depression level, anxiety level, stress level, OCD, facial muscle pain, disc displacement, degenerative joint diseases, and classified chronic pain (*p* < .05).

After fitting the research model by the logistic regression, as shown in Table 4, the variables of age and level of depression were the only variables present in the present study that affected the incidence of TMD and can help predict it.

**Table 4:** The results of fitting the research model using the logistic regression method.

Odds ratio	Significance value	Degree of freedom	Wald test	Standard deviation	Parameter estimation	Variable
0.492	0.269	1	1.224	0.641	−0.709	Regression constant
1.033	0.004	1	8.462	0.011	0.033	Age
	0.000	3	29.078			Depression level

To compare the probability of TMD in two groups with and without OCD, an independent two‐sample *t* test was used. According to Table 5, since the significance test statistic of equality of variances is reported to be 35.626 and the significance level is 0.000, it can be concluded that the chance of TMD is not the same among those with and without OCD. On the other hand, considering that both confidence intervals are negative, it can be concluded that the chance of TMD is higher among those with OCD than others.

**Table 5:** Average test of two independent populations.

95% confidence interval							
Inf	Sup	Standard error of the difference	Mean difference	Significance value	Degree of freedom	*T* test	
–0.0467	–0.2237	0.0450	–0.135	0.003	383	–3.004	Homogeneity of variance
–0.0463	–0.2241	0.0452	–0.135	0.003	366.350	–2.990	Inequality of variance

The results of Duncan's post hoc test in Table 6 show no significant difference in TMD between healthy individuals and those who only suffer from stress, but those who suffer from stress and OCD at the same time are at a higher risk of suffering from OCD and the incidence of TMD is more common.

**Table 6:** Duncan's post hoc test.

*α* = .05			
2	1	Num	Group
	0.2071	198	Normal
	0.3071	140	Stress only
0.4468		47	Stress and OCD
1.000	0.129		Significance value

Abbreviation: OCD, obsessive‐compulsive disorder.

## 5 DISCUSSION

The term TMD refers to a group of clinical conditions involving muscles of mastication, TMJ, and related structures or both. The exact etiology of most TMDs is unknown, and several hypotheses such as occlusal disharmony, excessive muscle activity, central pain mechanisms, psychological distress, and trauma have been proposed as the etiological factors of TMD.

There are many differences of opinion on the effective causes of TMD, which can be due to the multifactorial nature of TMD and the fact that, in general, comprehensive studies on all possible factors affecting TMD have been conducted less. This study investigated the effect of OCD and stress as factors affecting TMD. Due to the simultaneous presence of stress and OCD and the inability to separate the pure OCD group, we could not examine the effect of OCD on TMD in a separate group. As a result, we investigated the combination and association of these two to what extent it can increase the chance of TMD and whether this increase in risk is significant.

Since DASS42 was used to identify the stress level of individuals, in addition to obtaining the stress level, which is our main subject in this study, the level of anxiety and depression were also obtained, which were investigated as secondary and effective results. Among the limitations of this study, we can mention the number of cases due to the large number of questions in the questionnaires, fatigue of individuals at the end of the questions, and inconsistent answers or failure to answer the final questions can be observed.

The study results showed that the chance of TMD is 44.68% in those who suffer from stress and OCD at the same time and 24.85% in others, indicating that the chance of TMD among those who suffer from OCD and stress at the same time is 1.8 times that of others. It should be noted that stress does not affect the clinical symptoms of TMD but generally increases the chances of developing TMD, which is also true for stress and OCD. Although this study attempted to identify other factors affecting TMD, the results of logistic regression modeling showed that among the studied variables, only age and level of depression affected the probability of TMD.

Also, in this study, considering the fact that the included subjects were equalized in terms of not having developmental problems such as craniofacial syndromes, history of trauma, cross‐bite, clear occlusal interferences, class I and normal overjet and overbite, a more detailed investigation on the relationship between TMD and OCD was possible.

The results of the present study are consistent with the study of Sojka and colleagues who, in a study on the relationship between TMD and psychological factors in 324 students, reported a higher level of stress, anxiety, and depression in those with TMD (Sójka et al., 2019). Nguyen and colleagues investigated the relationship between bone changes in TMJ and stress and depression among the elderly and reported that TMJ changes were not associated with anxiety, depression, and limited TMJ function (Nguyen et al., 2019).

Since Costen (1934) stated his hypothesis about the improvement of patients with multiple jaw and ear problems after correcting the vertical dimension of occlusion, the relationship between occlusion disharmony and TMD was considered. The occlusion hypothesis was expanded to include other occlusal parameters in addition to vertical height loss that each seems to play a role in TMD (Costen, 1934). At the time of the report of Costen on the definite relationship between occlusal disorders and TMD and the improvement of TMD symptoms following occlusal treatments, patients with occlusal disorders and the symptoms of TMD were diagnosed as Costen's syndrome and had orthodontic treatment hoping that the correction of occlusal relationships would lead to the improvement of TMD symptoms. Today, no clear and convincing evidence exists for occlusal disharmony as a primary cause of TMD (Manfredini et al., 2017).

Overjet and overbite are not strongly related to joint click, crepitus, pain, and/or limited mouth opening (Michelotti et al., 2016). Although occlusal problems can be the starting factor in some cases, no significant difference has been reported in the occlusion of patients with myofascial pain and the control group. A systematic review (2012) showed inconsistent results in this field, mainly related to the lack of specific investigation of occlusal disorders. However, this systematic study showed that only a few occlusal disorders, such as open bite, excessive overjet, midline mismatch, and loss of five or more posterior teeth, are related to osteoarthritis (Türp & Schindler, 2012).

The mental disorder hypothesis suggests that TMD results from problems in an individual's functioning and generally occurs due to an individual's stressful environment and poor adaptive skills, leading to a disorder such as depression, anxiety, or both. Two hypotheses through which mental disorders can cause TMD have been proposed. The most common hypothesis is parafunctional habits that cause muscle pain over time. The second hypothesis states that mental disorders generally increase the risk of experiencing pain when faced with some phenomena, such as a traumatic yawn.

OCD is ranked as one of the 10 main causes of disability related to the disease and is characterized by persistent intrusive thoughts and repetitive behaviors. Although the neurobiological basis of OCD is still unknown, neuroimaging studies on those with OCD have shown evidence of excessive activity of the brain's orbitofrontal cortex, and the underlying cellular cause of this increased activity is not yet clear (Tanaka, 2021).

As mentioned, TMD relates to multiple etiological factors with social, cultural, and psychological components. Psychological factors of TMD can be divided into subgroups of behavioral symptoms such as bruxism, emotional symptoms such as stress, anxiety, and depression, and aspects related to memory (Barbosa et al., 2008). The challenge we often face is determining to what extent mental disorder is the cause or result of chronic pain. Psychological studies have shown that patients with TMD are similar to other chronic musculoskeletal pain disorders regarding psychological profile and psychological dysfunction (Bortsov et al., 2017).

An increase in chronic orofacial pain in TMD affects individuals' quality of life and health. As mentioned in previous studies, the most reported response to a stressful event is avoidant and intrusive thoughts (Fong & Loi, 2016). Stress, somatic distress, and depression may be potential etiological factors for the incidence of TMD‐related pain, and psychological factors are more evident and prominent in patients who experience chronic TMD pain (Heinen et al., 2017). In addition, it has been suggested that psychosocial factors can aggravate both pain behavior and the amount felt (Leary & Hoyle, 2009).

Psychological therapies aid self‐management by encouraging beneficial behaviors, minimizing potentially detrimental responses, and assisting in removing barriers to effective self‐management participation when appropriate. Psychological therapies may include reducing anxiety or depression, modifying stress reactivity or reducing habitual behaviors, introducing effective coping strategies, increasing confidence and ability to engage in rewarding and meaningful activities, redefining the meaning of pain, or redirecting focus away from pain and toward valued life goals (Fillingim et al., 2013).

Existing self‐management programs provided as part of routine care are highly variable and are frequently delivered without psychological training or support. There is some evidence that early psychological therapy aimed at individuals with higher reported dysfunction due to TMD can improve long‐term outcomes and lower the likelihood of persistent pain (Penlington et al., 2022).

Numerous studies have investigated the differences in OCD patients' management in the dental office. Dental patients with OCD exhibit a variety of OCD‐related obsessions or compulsives, which cause difficulties or changes in their dental operations. Compulsions or obsessions, being afraid of pathogens and contamination, aggressive thoughts toward others or self, having things symmetric in perfect sequence, excessive cleaning or handwashing, organizing things in a specific way, repeatedly checking things, and compulsive counting are symptoms reported in dental patients with OCD. Patients may become agitated if they are not often relieved throughout treatment sessions that everything around them, such as dental tools and chairs, is cleansed and disinfected (Chandna et al., 2014).

Another factor to consider in the management of TMD in OCD patients is the medications they are taking. Because consumption of fluoxetine or citalopram has been linked to increased bruxism in patients, consulting a psychiatrist and altering the medication regimen should be considered (Oulis et al., 2012).

Dentists who are the initial point of contact for individuals suffering from painful TMD are often unsure about psychological management elements. There is a need for updated training in psychological therapies for painful TMD to help clinicians and commissioners plan and create programs that best suit the demands of individuals with TMD and OCD.

## 6 CONCLUSION

According to the study results and data analysis and modeling by SPSS, it seems that stress and OCD can increase the risk of TMD symptoms by 1.8 times, which was determined after data analysis. This increase in risk is significant. Also, according to the positive correlation coefficient between TMD and both variables of stress and OCD, it can be concluded that increasing stress and OCD itself can be associated with increasing the probability of TMD. Therefore, it can be said that if stress is associated with OCD, it can increase the risk of TMD from 24.85% to 44.68%.

## AUTHOR CONTRIBUTIONS

Maryam Sadat Sadrzadeh Afshar and Behzad Salari conceptualized the idea of the study, and Ali Shobeiri and Kimia HafeziMotlagh contributed to the interpretation of data and the drafting, formatting, and editing of the manuscript. All authors read and agreed to the submitted version of the manuscript.

## CONFLICT OF INTEREST STATEMENT

The authors declare no conflict of interest.

## Data Availability

The data supporting this study's findings are available on request from the corresponding author.
